# Rigid Endoscope-Guided Endotracheal Intubation: An Alternative Technique for Anticipated Difficult Airway Management in Otolaryngology Patients

**DOI:** 10.7759/cureus.80643

**Published:** 2025-03-16

**Authors:** Ömer Bayır, Mehmet Murat Günay, Esma Altan, Güleser Saylam, Julide Ergil

**Affiliations:** 1 Otolaryngology - Head and Neck Surgery, Ankara Etlik City Hospital, Ankara, TUR; 2 Otolaryngology - Head and Neck Surgery, Lokman Hekim University, Ankara, TUR; 3 Anesthesiology, Ankara Etlik City Hospital, Ankara, TUR

**Keywords:** airway management, difficult airway, endoscopic intubation, endotracheal intubation, rigid endoscope

## Abstract

Rigid endoscope-guided endotracheal intubation is a valuable technique for managing difficult airways in ENT patients, especially those with airway masses or cancers in the oral cavity, oropharynx, larynx, and hypopharynx. Traditional methods, including laryngoscopy and videolaryngoscopy, may fail to visualize the larynx in such cases, necessitating alternative approaches. This technique can combine a rigid endoscope with an endotracheal tube to provide clear visualization of the vocal cords with the trachea and facilitate intubation. This method can reduce the risk of airway trauma by allowing precise control and visualization throughout the procedure. It can also eliminate the need for blind insertion, improving safety. The technique can be effective in both anticipated and emergency difficult airway management scenarios, reducing the need for invasive procedures such as tracheotomy. This technique offers a promising alternative to traditional methods, especially for patients not candidates for awake intubation.

## Introduction

A difficult airway is characterized by challenges or failure in one or more of the following steps: mask ventilation, laryngoscopy, supraglottic airway ventilation, tracheal intubation, extubation, or invasive airway management, either anticipated or unanticipated, even when performed by trained anesthesia professionals [1]. Difficult intubation refers to scenarios requiring multiple attempts or failure despite repeated attempts [1]. Cattano et al. report that the incidence of difficult intubation in the adult population ranges from 1.2% to 3.8%, while the occurrence of difficult mask ventilation is estimated to be between 0.01% and 0.5% [2]. Difficult intubations are even more commonly encountered in otolaryngologic practice, particularly in patients with malignancies of the oral cavity, oropharynx, larynx, and hypopharynx [3]. In these patients, visualization of the larynx may be impossible even with direct or video laryngoscopy. Due to the underlying pathology, mask ventilation or laryngeal mask ventilation may also be challenging, necessitating invasive airway procedures such as tracheotomy or cricothyrotomy, which significantly increase patient morbidity and mortality [3]. Iseli et al. recommend that, to reduce morbidity and mortality in patients with head and neck cancer, a secondary airway plan should always be prepared, with essential involvement from head and neck surgeons [4]. In our practice, we utilize rigid endoscope-guided endotracheal intubation to manage difficult airways in pediatric and adult patients. This technical report aims to describe rigid endoscope-guided endotracheal intubation as an effective method for anticipated difficult airway management.

## Technical report

**Figure 1:**
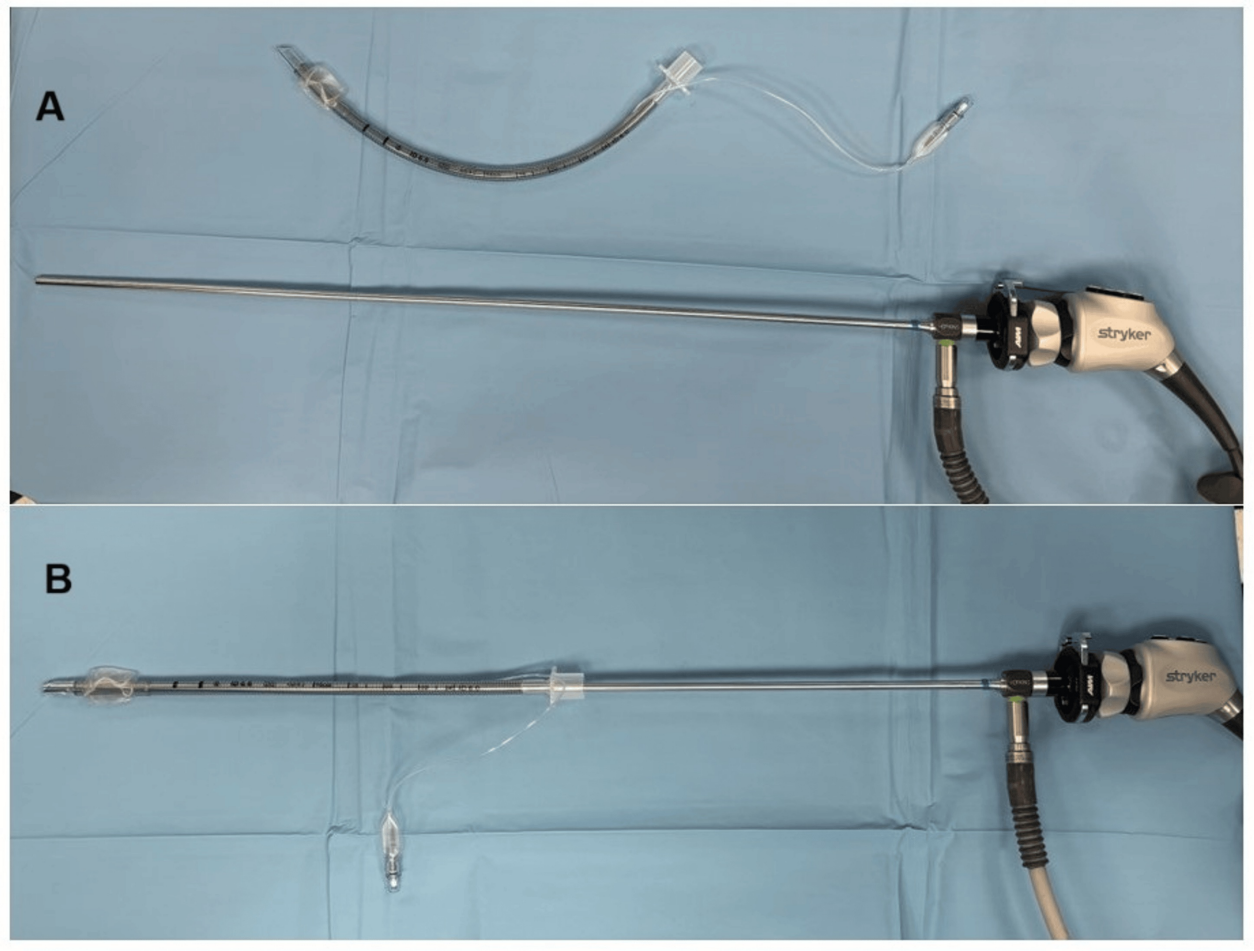
Preparation of a rigid endoscope with a cuffed endotracheal tube (A) A cuffed endotracheal tube and a rigid endoscope are prepared. (B) The cuffed endotracheal tube is placed over a 5 mm x 302 mm rigid endoscope. Subsequently, the examination of the pathology narrowing the airway and the intubation procedure are performed.

Standard monitoring was performed under operating room conditions, including electrocardiography, non-invasive blood pressure, and oxygen saturation (pulse oximetry). Following preoxygenation with 100% oxygen, general anesthesia was induced using 2 mg/kg propofol and 1 mcg/kg fentanyl. Rocuronium (0.6 mg/kg) was administered as a muscle relaxant after confirming successful face mask ventilation. After rocuronium administration, patients were ventilated with 100% oxygen via mask for three minutes. All necessary intubation equipment was prepared before induction. A cuffed or uncuffed endotracheal tube, appropriate for the size of pediatric patients, was positioned over the 2.7 mm/4 mm x 18 cm 0° rigid endoscope. For adult patients, a size-appropriate cuffed endotracheal tube was placed over a 5 mm x 30 cm 0° rigid endoscope (HOPKINS® telescope, KARL STORZ, Germany) (Figure 1).

**Figure 2:**
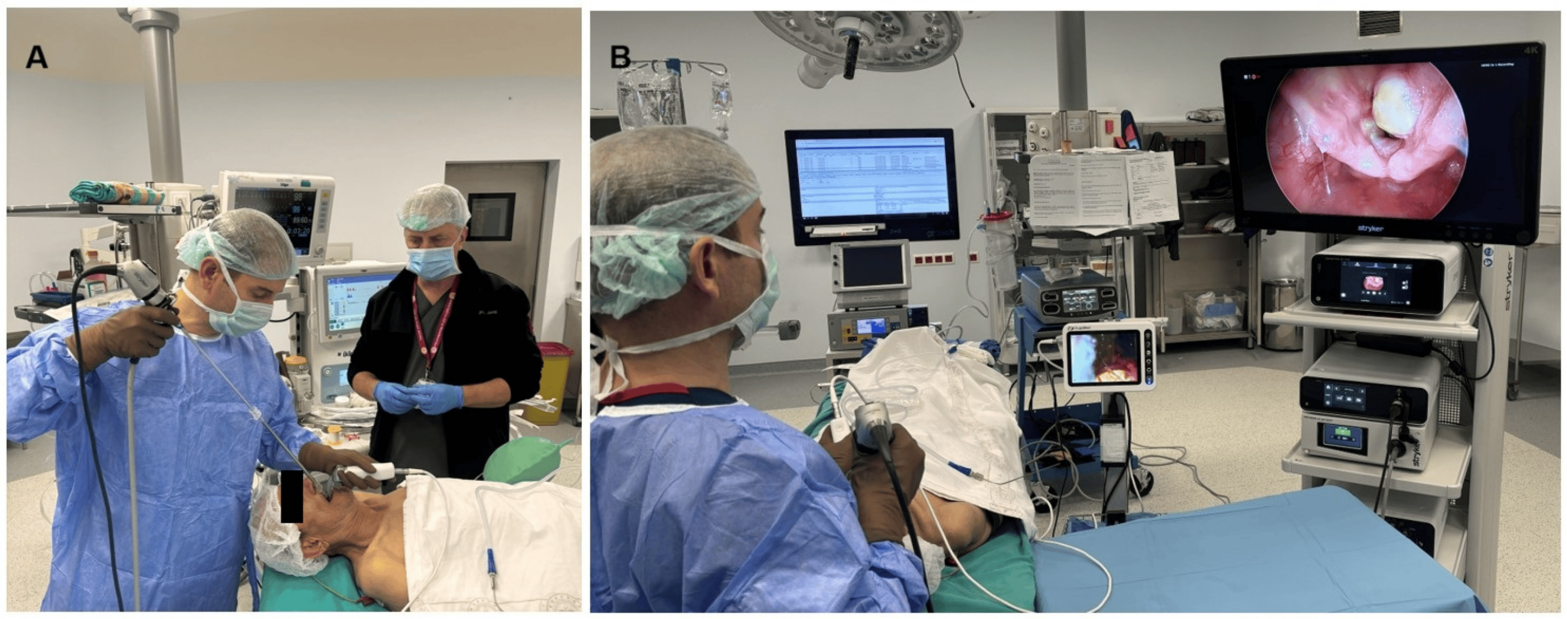
Pathology causing the difficult intubation examination with the tube-mounted endoscope (A) The tube-mounted endoscope is advanced orally with the right hand to the glottis level, visualizing the vocal cords. (B) The pathology causing difficult intubation is examined, and then the endoscope is advanced past the pathology into the trachea. The tube is then left in the trachea while the endoscope is withdrawn.

A lubricant can be applied during this procedure to easily remove the endotracheal tube from the endoscope. The rigid endoscope was connected to a video camera monitor system, and the endoscopic image was transmitted to a high-quality monitor. The patient was positioned in a sniffing position. A direct laryngoscope in the left hand (a video laryngoscope could also be used) was used to retrace the right half of the tongue and the base. The tube-mounted endoscope was then advanced orally with the right hand to the glottic level, visualizing the vocal cords (Figure 2).

At this stage, the pathology contributing to difficult intubation was also assessed. Once the subglottic area was traversed and a safe zone was reached, the assistant disengaged the endotracheal tube from the endoscope, completing the intubation. Correct endotracheal tube placement was confirmed through chest inspection, auscultation of both lungs' upper and lower poles with a stethoscope, and detection of CO₂ on the capnogram.

## Discussion

Preoperative airway evaluation and identifying patients with difficult airways are critical for effective airway management. Upper airway pathologies and anatomical abnormalities can be assessed using physical examination methods and, if necessary, imaging techniques [1,3]. Additionally, preoperative flexible endoscopy provides valuable insights into the patient's upper airway pathology [1,5].

Following a comprehensive preoperative evaluation, essential airway management equipment should be readily available, patients should be preemptively informed, preoxygenation should be performed, and patient positioning and sedation should be individualized. Furthermore, personnel experienced in managing difficult intubation should be present or readily accessible [1].

Awake endotracheal intubation remains the preferred approach for anticipated difficult airway management, with success rates ranging from 88% to 100% [1]. Several non-invasive tools are also employed in difficult airway management, including rigid laryngoscopic blades of varying sizes and designs, bougies, stylets, lighted stylets, video laryngoscopes, flexible endoscopes, supraglottic airway devices, and rigid bronchoscopes [1,6]. However, there remains a lack of consensus in the literature regarding the most effective noninvasive device or the preferred approach in cases of failed intubation or anticipated difficult airway scenarios.

In our practice, awake intubation is preferred in cooperative patients with difficult airways. However, for patients uncooperative with awake intubation and in cases where preoperative endoscopic evaluation reveals difficulty in glottic visualization, we employ the "rigid endoscope-guided endotracheal intubation" method. Additionally, this method has been successfully utilized in unexpected and emergency airway management scenarios in the operating room.

Rigid endoscopes, commonly used by otolaryngologists and head and neck surgeons in airway surgeries, are longer and thicker than standard nasal endoscopes, providing a superior field of view, improved magnification, and better resolution for tracheal access. When used orally, they cause minimal trauma, require fewer additional skills, and have a shorter learning curve, facilitating ease of use. These characteristics make them a practical tool for intubation.

Intubations performed with a stylet or bougie are often blind insertions where the device tip is not directly visible, increasing the risk of airway trauma [7]. However, in rigid endoscope-guided endotracheal intubation, the endotracheal tube is advanced under direct vision, minimizing the risk of upper and lower airway trauma. Additionally, advancing the tube into the trachea under continuous endoscopic guidance, with real-time visualization on a monitor, enhances intubation success rates. Furthermore, this technique allows for the detailed assessment of difficult airway pathologies and tumor mapping. However, this approach may not be suitable in cases of severe trismus and conditions where neck extension is severely restricted, such as ankylosing spondylitis.

## Conclusions

The rigid endoscope-guided endotracheal intubation technique represents a safe and effective approach for patients with difficult airways, particularly those with airway masses who are not cooperative with awake intubation. This method can be successfully employed in the operating room for unexpected emergencies and difficult airway management, potentially eliminating the need for invasive procedures such as tracheotomy or cricothyrotomy.
